# Crystallographic order and decomposition of [Mn^III^_6_Cr^III^]^3+^ single-molecule magnets deposited in submonolayers and monolayers on HOPG studied by means of molecular resolved atomic force microscopy (AFM) and Kelvin probe force microscopy in UHV

**DOI:** 10.1186/1556-276X-9-60

**Published:** 2014-02-05

**Authors:** Aaron Gryzia, Timm Volkmann, Armin Brechling, Veronika Hoeke, Lilli Schneider, Karsten Kuepper, Thorsten Glaser, Ulrich Heinzmann

**Affiliations:** 1 Molecular and Surface Physics, Bielefeld University, Universitaetsstr 25, Bielefeld 33615, Germany; 2 Inorganic Chemistry, Bielefeld University, Universitaetsstr 25, Bielefeld 33615, Germany; 3 Elektronische Struktur (Experiment), Osnabrueck University, Barbarastrasse 7, Osnabrueck 49069, Germany

**Keywords:** AFM, KPFM, Single-molecule magnet, HOPG, Ordered monolayer, Decomposition, UHV

## Abstract

Monolayers and submonolayers of **[Mn**^**III**^_**6**_**Cr**^**III**^**]**^**3+**^ single-molecule magnets (SMMs) adsorbed on highly oriented pyrolytic graphite (HOPG) using the droplet technique characterized by non-contact atomic force microscopy (nc-AFM) as well as by Kelvin probe force microscopy (KPFM) show island-like structures with heights resembling the height of the molecule. Furthermore, islands were found which revealed ordered 1D as well as 2D structures with periods close to the width of the SMMs. Along this, islands which show half the heights of intact SMMs were observed which are evidences for a decomposing process of the molecules during the preparation. Finally, models for the structure of the ordered SMM adsorbates are proposed to explain the observations.

## Background

Molecular magnetism has become a vast subject for investigation from the field of coordination chemistry and physics [1-4]. Single-molecule magnets (SMMs) are under research regarding several future applications such as quantum computing [5], magnetic refrigeration [6,7], and high-density information storage [8]. The control of properties, particularly with regard to the interaction of the SMMs with their environment is crucial for its application, including of course the adsorption onto surfaces and the stability of the SMMs. So far structured application of SMMs has been performed using microcontact printing [9] or by functionalization of the SMMs with surface-active groups (e.g., thiol groups), ensuring a self-organizing process on the surface resulting in ordered SMM structures [10].

The designed SMM [{(talen^*t*-Bu^_2_)Mn^III^_3_}_2_{Cr^III^(CN)_6_}]^3+^ (**[Mn**^**III**^_**6**_**Cr**^**III**^**]**^**3+**^) with H_6_talen^*t*-Bu^_2_ = 2,4,6-tris(1-(2-(3,5-di-*tert*-butylsalicylaldimino)-2-methylpropylimino)-ethyl)-1,3,5-trihydroxybenzene consisting of six Mn^III^ and one Cr^III^ ion exhibits a ground state of *S*_t_ = 21/2 with a significant easy-axis type magnetic anisotropy. This results in an energy barrier for spin reversal. The C_3_ symmetry minimizes the quantum tunneling through this energy barrier so that a slow relaxation of the magnetization can be observed at low temperatures with a blocking temperature around 2 K [11-15].

**[Mn**^**III**^_**6**_**Cr**^**III**^**]**^**3+**^ is a triple-charged cation. Salts of **[Mn**^**III**^_**6**_**Cr**^**III**^**]**^**3+**^ with different monoanionic counterions (*X* = BPh_4_, PF_6_IOAc, ClO_4_, lactate) and the trianionic counterion [Cr(CN)_6_)]^3-^ have been prepared so far. X-ray crystallography measurements of this molecule show a height of 1.22 nm and a width of 2.13 nm.

The oxidation state of the manganese atoms of **[Mn**^**III**^_**6**_**Cr**^**III**^**]**^**3+**^ stays intact when prepared on the surface (e.g., gold, highly oriented pyrolytic graphite (HOPG)) [16]. Nevertheless, X-ray absorption measurements have shown different radiation sensitivities depending on the anion used in which (ClO_4_)^-^ anions appeared to be one order of magnitude more stable than tetraphenylborate and lactate [17].

The arrangement of the adsorbed molecules of **[Mn**^**III**^_**6**_**Cr**^**III**^**]**^**3+**^ depends heavily on the substrate used. HOPG allows the SMMs to form islands of monolayers, whereas on substrates like Si, the formation of hemispheric clusters on the surface has been observed [18].

The characterization of the topology of adsorbed **[Mn**^**III**^_**6**_**Cr**^**III**^**]**^**3+**^ SMMs was performed by means of nc-AFM [19-21]. Further information was gained by frequency modulated Kelvin probe force microscopy (FM-KPFM) in order to measure the local contact potential differences (LCPD).

## Methods

The molecules observed in the study were **[Mn**^**III**^_**6**_**Cr**^**III**^**]**(ClO_4_)_3_. The substrates used were HOPG. The methods used in this study were non-contact atomic force microscopy, Kelvin probe force microscopy, and X-ray photoelectron spectroscopy.

A solution of 10 μl of **[Mn**^**III**^_**6**_**Cr**^**III**^**]**(ClO_4_)_3_ solved in methanol in order to achieve a concentration of 1 × 10^-5^ mol/l was prepared. This solution was applied in air at room temperature onto a 10 × 10 mm^2^ HOPG (NT-MDT, ZYB quality, Zelenograd, Moscow, Russia) surface, using the droplet technique [22,23]. The HOPG substrate was glued onto the surface of Omicron Carriers (Omicron NanoTechnology, Taunusstein, Germany) and tilted at an angle of 57° to the horizontal plane in order to achieve a more homogeneous wetting. The number of molecules applied is sufficient for approximately one monolayer. The sample was put inside the load lock of the ultra-high vacuum (UHV) apparatus immediately following deposition of the solution with the molecules. The SMM molecules adsorbed on the HOPG surface by this procedure stay intact with respect to the composition, magnetic properties, and their oxidation state, as was confirmed earlier using XAS [16,17] and X-ray photoelectron spectroscopy (XPS) [18]. Experiments were performed with a modified Omicron UHV AFM/STM in non-contact mode at room temperature (approximately 22°C) and a pressure of 3 × 10^-8^ Pa. The self-oscillating mode was replaced by a phase locked loop (PLL) setup from Nanosurf (easyscan2, Nanosurf, Woburn, MA, USA). The sensitivity and the signal-to-noise ratio were increased by replacing the internal laser diode by an external one combined with a replacement of the preamplifier of the position-sensitive diode signal, according to the concept of Torbrugge et al. [24].

The setup with FM-KPFM [25] using a lock-in amplifier (Signal Recovery, Oak Ridge, TN, USA) in conjunction with a proportional integral (PI) controller (Stanford Research Systems, Sunnyvale, CA, USA) in order to analyze the local contact potentials of the SMM. Silicon cantilevers (NSC15, MikroMasch, San Jose, CA, USA) with a resonance frequency of 325 kHz and a radius at the apex of 10 to 15 nm were used for the measurements. Cantilevers were sputtered with an ion setup in order to clean any adsorbed contamination of the tip. Z calibration was carried out by measuring monoatomic step edges of HOPG. The KPFM measurements were realized with an applied ac current of 1.3 kHz and an amplitude of 1 V in order to increase the contrast of different LCPD regions [26]. The setup has proven atomic resolution on KBr both in the topographic as well as in the LCPD mode.

The chemistry of **[Mn**^**III**^_**6**_**Cr**^**III**^**]**^**3+**^ in solution was studied by electrospray ionization mass spectrometry (ESI-MS), ultraviolet–visible near infrared (UV–vis-NIR) absorption spectroscopy, and electrochemistry [15].

**Figure 1:**
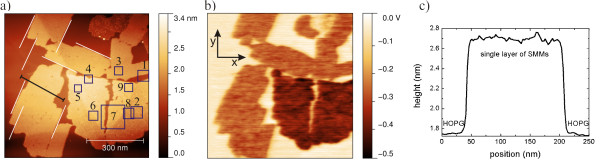
**Nc-AFM micrograph of [Mn**^**III**^_**6**_**Cr**^**III**^**](ClO**_**4**_**)**_**3 **_**on HOPG, 753 × 790 nm**^**2 **^**scan.** The substrate is covered 60% with a monolayer. Many of the monolayer's edges run parallel to each other. **(a)** Topography with nine areas named from 1 to 9. **(b)** LCPD shows two main areas: one with a LCPD of -0.26 V for the brighter islands and one with a LCPD of -0.38 V in the bottom right quadrant of the image. **(c)** Line scan across an island. The position of the line scan is marked with a black line in **(a)**.

The nomenclature of the directions, *x* and *y*, in an image is depicted in the XY-coordinates in Figure 1b and is valid for topography and LCPD images. The color scale for the topographic heights of the images each is chosen for maximized contrast. LCPD data is presented relative to the level of HOPG.

## Results and discussion

### Crystallographic order of [Mn^III^_6_Cr^III^](ClO_4_)_3_ monolayer

Islands of **[Mn**^**III**^_**6**_**Cr**^**III**^**]**(ClO_4_)_3_ covering 30% to 60% of the HOPG surface, depending on the scan position, were observed. The islands show heights of about 1 nm and exhibit flat top structures. Beside the topography channel, the uncovered HOPG surface and the islands show different LCPD.

**Figure 2:**
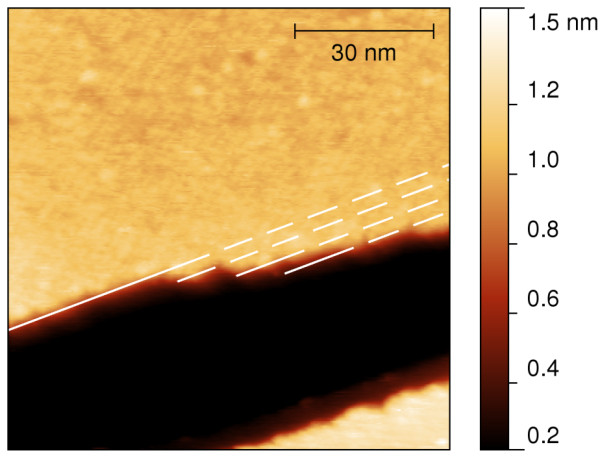
**Nc-AFM micrograph of [Mn**^**III**^_**6**_**Cr**^**III**^**](ClO**_**4**_**)**_**3 **_**on HOPG 94 × 99 nm**^**2 **^**scan of area 1 in Figure**1**.** Several molecular kinks occur along an edge of an island of the SMMs.

The islands are discriminated by the LCPD and by their internal structure. Figure 1 shows islands with heights of 1 nm (Figure 1c) covering 60% of the surface. The corresponding KPFM image (Figure 1b) discriminates between islands with a LCPD of -0.26 and -0.38 V. The latter is in the bottom right part of the image and is a single island with a rip which nearly cuts the island in half. Important to note is that several edges of these islands run parallel to each other. Furthermore, kinks occur along the edges which fit the size of one molecule. Figure 2 shows area 1 of Figure 1 showing these kinks.

The island in the lower right part of Figure 1 shows a stripe-like texture along the whole area and a LCPD of -0.38 V. The period of these stripes is in the order of 2.9 ± 0.2 nm and keeps its orientation along the whole island. Obviously, the distance of the parallel lines is larger than the distance between single molecules with a size of 2.13 nm along the lines.

**Figure 3:**
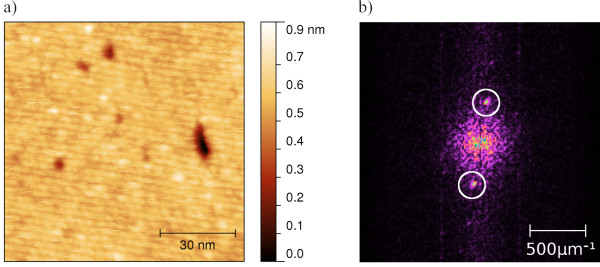
**Nc-AFM micrograph of [Mn**^**III**^_**6**_**Cr**^**III**^**](ClO**_**4**_**)**_**3 **_**on HOPG, 94 × 99 nm**^**2 **^**scan.** The scan was done in area 2 of Figure 1 with a LCPD of -0.38 V. **(a)** Topography showing stripes which cover the whole area of -0.38 V. **(b)** FFT image revealing a period of the stripes of 2.9 ± 0.2 nm.

Figure 3a shows the enlargement of the area 2 exhibiting the stripe structure interrupted only by holes of few nanometers in size which do not influence the progression of the texture. In the corresponding fast Fourier transformation (FFT) image in Figure 3b, the twofold symmetry is seen.

**Figure 4:**
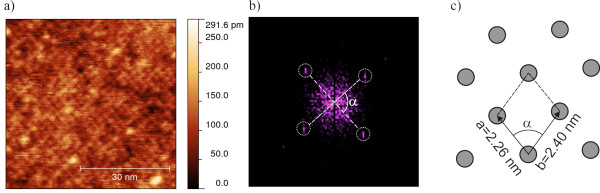
**Nc-AFM micrograph of [Mn**^**III**^_**6**_**Cr**^**III**^**](ClO**_**4**_**)**_**3 **_**on HOPG.** Scan range, 57 × 59 nm^2^ of area 3 in Figure 1. **(a)** The area is fully covered with SMMs and shows a crystallographic order. **(b)** FFT of the image revealing four spots indicating two predominant directions of the lattice. **(c)** Real space model of the elementary unit cell of the lattice.

In the layer of area 3 shown in Figure 4a, the symmetry of the SMM layer appearing shows not just two spots in the corresponding FFT in Figure 4b but four. The adsorption of the SMM on the surface is depicted in Figure 4c using a real space model. Two periods in the range of 2.26 ± 0.20 and 2.40 ± 0.19 nm very close to the size of the molecule (2.13 nm) are observed. The lattice shows a symmetry which is twofold but close to a fourfold one within the error bars given. Furthermore, the difference in the texture of the layers corresponding to Figures 3 and 4 is found in the LCPD image of Figure 1b. The area of Figure 3 originates from the bottom right quadrant of Figure 1, exhibiting a LCPD of -0.38 V in contrast to the remaining islands with a LCPD of -0.26 V.

The angle *α* between the reflexes shown in Figure 4b is described by 83° ± 7° which is also close to a fourfold symmetry within the error bars. The texture is visible at every position in the image and keeps its periods and angles. In our case, a transformation from Fourier to real space and vice versa does not change the relative angle between two pairs of spots. The orientations of the areas 2 and 4 to 9 of Figure 1 are identical to each other within the error of ±7° which is a strong indication for a commensurate adlayer structure along the crystallographic order of the substrate.

On the one hand, the lattice constant of the 2D structure with the close to fourfold symmetry (Figure 4) matches the size of the SMM. On the other hand, the lattice constant of the 1D structure (2.9 nm) is significantly higher than the SMMs' size over large range. Although no preferred orientation was observed, the driving force for the latter structure is very much likely caused by a stronger interaction of the SMM with the substrate compared with the 2D structure.

### Model of the adsorption of [Mn^III^_6_Cr^III^](ClO_4_)_3_ on top of HOPG

**Figure 5:**
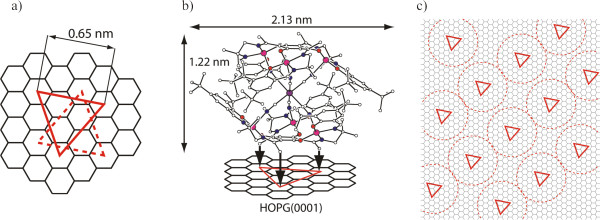
**Model of adsorption sites. (a)** Adsorption sites of **[Mn**^**III**^_**6**_**Cr**^**III**^**]**^**3+**^ on HOPG. **(b) [Mn**^**III**^_**6**_**Cr**^**III**^**]**^**3+**^ adsorbs on HOPG with its methyl groups fitting exactly the shown sites forming an equilateral triangle. **(c)** Model of the lattice of **[Mn**^**III**^_**6**_**Cr**^**III**^]^**3+**^ on HOPG matching our data with respect to the angle and periods. The circles illustrate the molecule's size measured in crystal [30].

**[Mn**^**III**^_**6**_**Cr**^**III**^**]**^**3+**^ has, besides others, three methyl groups at the top and three at the bottom. These three methyl groups span a plane perpendicular to the vertical axis of the SMM. The methyl groups are assumed to bind to the HOPG surface by C-H/*π* interactions. The binding is suggested to be of hollow site type which is supported by own calculations and consistent with [27-29]. The distance of the three methyl groups to each other is 0.65 nm [30] leading to two orientations in which the SMM can adsorb to hollow site positions on HOPG as depicted with the red equilateral triangle in Figure 5a,b.

This gives us a model which depends on four variables. These are to match the acquired datasets consisting out of three parameters: the two periods and the angle between them. The best fit received is shown in Figure 5c. In this model, we have two periods, 2.28 and 2.34 nm, and an angle between the orientations of 87.2° which is in agreement with the experimental results, within their uncertainties.

The lack of observation of SMM stacking and Volmer-Weber growth when using (ClO_4_)^-^ as anion implies a stronger interaction between the substrate and the SMM than between two SMMs. In the case of the texture shown in Figure 3, a stronger SMM-substrate interaction than that inside the layer of Figure 4a must take place because the orientation of the texture is kept over an area of 0.125 μm^2^ whereby the area is almost fully separated in two islands as given in Figure 1.

### Islands of SMMs with half the height of full ones

**Figure 6:**
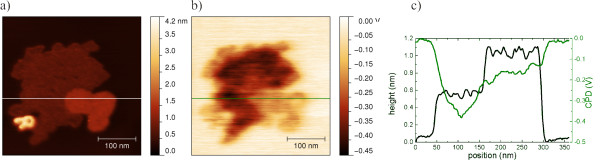
**Nc-AFM-micrograph of islands of [Mn**^**III**^_**6**_**Cr**^**III**^**](ClO**_**4**_**)**_**3 **_**on HOPG, 359 x 377 nm**^**2 **^**scan.** Islands with heights of 0.5 nm, 1.0 nm, and a cluster with 4 nm can be observed. **(a)** Topography. **(b)** LCPD. **(c)** Line scan of the nc-AFM image (topography, black; white line in **(a)**; LCPD, green).

We observe structures resembling islands of monolayers of **[Mn**^**III**^_**6**_**Cr**^**III**^**]**(ClO_4_)_3_ with a height of 1.0 ± 0.1 nm as given in Figure 1c. Besides these heights, we also found islands at other positions outside Figure 1 with just approximately half the height of a SMM, 0.50 ± 0.05 nm. Figure 6 shows an island covering 29% of the image with a height of 0.5 nm and a second island covering 7% of the image with a height of 1 nm. In addition, a cluster of molecules with a height of over 4 nm occurs which exhibits no internal structure. Even higher clusters have been observed on silicon substrates [18]. In the line scan of Figure 6c, the heights of two islands are shown. While preserving sharp edges, distinct heights can be observed for the higher and lower islands with 1.0 and 0.5 nm, respectively. Both islands reveal a flat structure on top.

The corresponding LCPD (Figure 6b) shows a significant change in the contrast of the two islands with regard to HOPG. The line scan is plotted in Figure 6c in green. The higher islands with values up to -0.23 V give a lower contrast in their LCPD than the lower islands with maximal values of -0.45 V with respect to HOPG.

**Figure 7:**
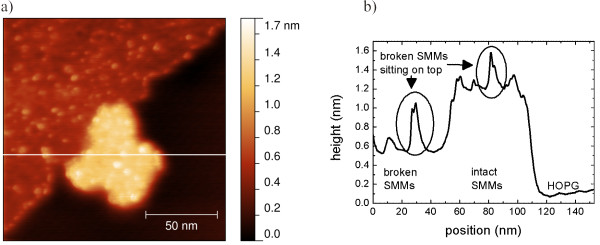
**Nc-AFM-micrograph of an island of [Mn**^**III**^_**6**_**Cr**^**III**^**](ClO**_**4**_**)**_**3 **_**on HOPG, 153 × 160 nm**^**2 **^**scan. (a)** An island with a height of 1.1 nm in contact with a lower island of broken molecules where single fragments are deposited on top of both islands.** (b)** Line scan of the nc-AFM image.

Small elevations can be found on top of layers with full and half the height of a single SMM. Figure 7 shows islands with such elevations with diameters smaller than 5 nm and heights up to 0.4 nm.

### Model of molecules with full and half the height on HOPG

The two different heights can be assigned to the following states:

• The areas with a height of approximately 1 nm are caused by **[Mn**^**III**^_**6**_**Cr**^**III**^**]**(ClO_4_)_3_. The molecules seem to be intact.

• The areas with half the height of a SMM refer to molecules with a changed composition.

**Figure 8:**
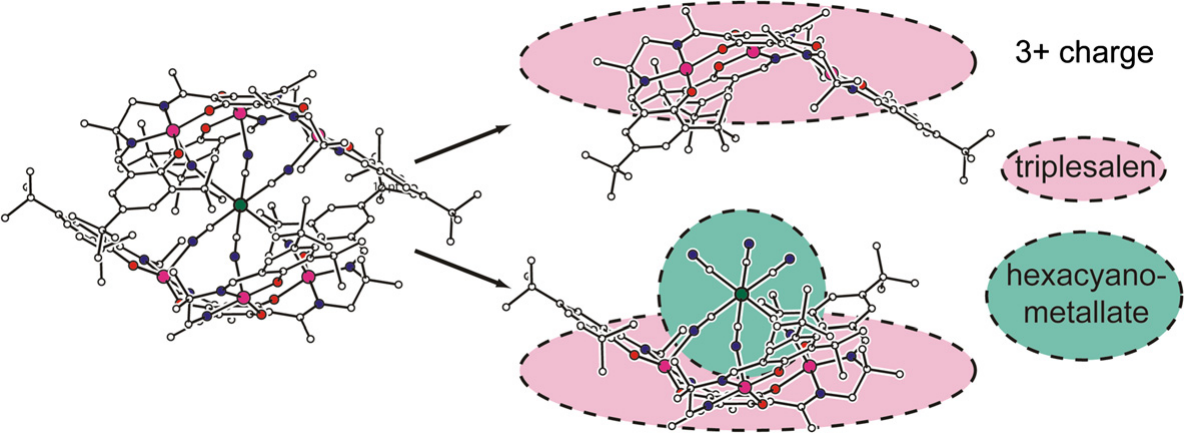
**Model of [Mn**^**III**^_**6**_**Cr**^**III**^**]**^**3+ **^**breaking into its building blocks.** This leaves one triplesalen with a 3+ charge and one neutral triplesalen-hexacyanometallate complex. Each SMM is surrounded by three tetraphenylborate counterions which are not depicted in this figure.

The way **[Mn**^**III**^_**6**_**Cr**^**III**^**]**(ClO_4_)_3_ adsorbs to the surface of HOPG indicates that the lateral dimensions cannot be changed. This means that the dipole moment of the two kinds of adsorbates must differ from each other. Due to the molecule being a three-cation, a change in the dipole moment must be caused by a decomposition of the SMM. In our model depicted in Figure 8, the SMM breaks into its building blocks consisting of one triplesalen with a remaining 3+ charge and a triplesalen still bonded to the hexacyanometallate of a 3- charge. The complex of the triplesalen and the hexacyanometallate is neutral. These molecules are the pre-stage for synthesizing **[Mn**^**III**^_**6**_**Cr**^**III**^**]**^**3+**^ which proves that such a decomposition is possible without the stability of the remaining components being destroyed. Furthermore, this increases the likeliness that the SMM breaks into its pre-stage components and not in other compositions. Decompositions are common on surfaces in catalytic processes [31-33] and have been observed with C_60_[34] but not yet with SMMs on HOPG. To date, it is just known only that SMMs and other large molecules in general may decompose over time [35]. Thus, the features on top of the islands with intact and broken SMMs can be assigned to fragments of the SMM. This leads to two remaining parts of the SMM, a bigger neutral molecule (triplesalen + hexacyanometallate) and a triple positive-charged triplesalen ion both being adsorbed on top of HOPG. Thus, the anions attach themselves to the single triplesalen in order to neutralize the remaining charge of the system. The heights of the observed structures match this description.

The dipole moment *μ* of an adsorbate on top of a surface is calculated using the LCPD ∆*Ф*, *σ* as the density of the adsorbates at the surface and *ε*_0_ as vacuum permittivity to: $µ=\frac{\varepsilon_{0}\mathrm{\Delta Ф}}{\sigma }$.

With a constant surface density of the adsorbates of one molecule per (2.5 nm)^2^, the resulting dipole moments are -1.94 × 10^-29^ Cm for the single-triplesalen complex with 0.5-nm height and -9.96 × 10^-30^ Cm for the intact SMM with 1-nm height. We have not yet observed the anions directly, but their occurrence close to the molecule is obvious. Without the anions, the positive charges of the broken molecules, which are delocalized in the intact molecule, should feature a distance to the surface of about 40 pm. As this is not possible, the molecules must be surrounded by the anions diminishing the dipole moment. XPS measurements confirm the stoichiometry of the SMM and its anions after preparation on the surface. ESI-MS, UV–vis-NIR absorption spectroscopy, and electrochemistry provide no evidence for a partial decomposition of **[Mn**^**III**^_**6**_**Cr**^**III**^**]**^**3+**^ into its three molecular building blocks in solution. However, an only minor decomposition cannot be ruled out. Therefore, it appears more likely that the decomposition observed here is supported by interaction with the surface.

## Conclusions

We have shown **[Mn**^**III**^_**6**_**Cr**^**III**^**]**(ClO_4_)_3_ adsorbing on top of HOPG and creating a 2D array and developed a corresponding model of the lattice. This model matches the observed features and explains the twofold structure of the superlattice, the angles, and the observed periods.

Furthermore, we have found layers with just half the height expected for intact molecules and identified them as broken SMMs which have become decomposed into pre-stages of the molecule. We have developed a model of how the intact and broken molecules adsorb to the substrate.

## Competing interests

The authors declare that they have no competing interests.

## Authors’ contributions

AG and TV carried out the AFM measurements supervised by AB and UH. LS and KK carried out the XPS measurements supervised by KK. VH synthesized the SMMs supervised by TG. All authors read and approved the final manuscript.
